# Research on Fatigue Life Prediction Method of Spot-Welded Joints Based on Machine Learning

**DOI:** 10.3390/ma18153542

**Published:** 2025-07-29

**Authors:** Shanshan Li, Zhenfei Zhan, Jie Zou, Zihan Wang

**Affiliations:** 1 School of Mechanical and Electrical and Vehicle Engineering, Chongqing Jiaotong University, Chongqing 400074, China; 13837043691@163.com (S.L.); zoujie@mat-jitri.cn (J.Z.); 15833861595@163.com (Z.W.); 2 Materials Bigdata and Applications Division, Materials Academy Jitri, Suzhou 215131, China

**Keywords:** spot-welding joints, fatigue life prediction, machine learning, structural stress method, random forest

## Abstract

Spot-welding joints are widely used in modern industries, and their fatigue life is crucial for the safety and reliability of structures. This paper proposes a method for predicting the fatigue life of spot-welding joints by integrating traditional structural stress methods and machine learning algorithms. Systematic fatigue tests were conducted on Q&P980 steel spot-welding joints to investigate the influence of the galvanized layer on fatigue life. It was found that the galvanized layer significantly reduces the fatigue life of spot-welding joints. Further predictions of fatigue life using machine learning algorithms, including Random Forest, Artificial Neural Networks, and Gaussian Process Regression, demonstrated superior prediction accuracy and generalization ability compared to traditional structural stress methods. The Random Forest algorithm achieved an R^2^ value of 0.93, with lower error than traditional methods. This study provides an effective tool for the fatigue life assessment of spot-welding joints and highlights the potential application of machine learning in this field.

## 1. Introduction

Spot-welding joints, a key connecting component in modern industries, are widely used in automotive, aerospace, shipbuilding, and other fields, especially in applications involving high-strength steel. With the extensive use of high-strength steels (such as Q&P980 steel) and galvanized steels in industries, the prediction of the fatigue life of spot-welding joints has become an important research topic to ensure structural safety and reliability. The fatigue performance of spot-welding joints is not only affected by factors such as the properties of welding materials, welding processes, and joint configurations but also closely related to factors such as thermal loads and stress states in the external environment. Therefore, accurately predicting the fatigue life of spot-welding joints is of great significance for improving the reliability and safety of structural design, manufacturing, and maintenance [1].

To further investigate the fatigue performance of spot-welding joints, this study conducted systematic fatigue tests on Q&P980 steel spot-welding joints. Q&P980 steel, known for its high strength and toughness, is widely used in automotive manufacturing and other engineering structures. Zou et al. [2] investigated the effects of liquid metal embrittlement (LME) cracks on the strength and fatigue life of spot-welded joints of galvanized Q&P980 steel using tensile–shear (TS) specimens but did not cover other specimen types. This study further extends their work by adding cruciform pull (CP) specimens. The fatigue tests were performed using constant amplitude load control. The results showed that the galvanized layer significantly reduced the fatigue life of the spot-welding joints, especially under the TS loading mode. In contrast, under the CP loading mode, the difference in fatigue life between galvanized and non-galvanized specimens was relatively small. These test results provided important data support for the subsequent fatigue life prediction model.

In terms of fatigue life prediction for spot-welding joints, traditional methods mainly rely on structural stress analysis to estimate the fatigue life of the joints. These methods predict the fatigue life of a welded joint by calculating the film and bending stresses at the edges of the welded joint and correlating them with the welded joint fatigue data. For example, the structural stress calculation method proposed by Rupp et al. [3] in 1995 treats the weld joint as a disk model and derives the structural stresses by calculating the forces and moments in the three principal stress directions in the local coordinate system of the weld joint. The region with the largest stress variance on the circumference of the weld joint is considered a potentially hazardous point, and the structural stress range at this point is used as a fatigue control parameter to predict the fatigue life of the spot-welded joint. However, these methods have certain limitations in practical applications, especially when dealing with high-strength and galvanized steels. This is because they ignore the nonlinear behavior of materials and various complex influencing factors, which often limit their prediction accuracy. In particular, during the welding of galvanized steels, the galvanized layer may induce liquid metal embrittlement (LME), a phenomenon that significantly accelerates crack propagation in the joints and thus greatly affects their fatigue performance [4]. Therefore, predicting the fatigue life of spot-welding joints in galvanized steels has become an important issue that urgently needs to be resolved in the field of welding technology.

With the rapid development of computer science and machine learning technology, machine learning methods have been widely used in many engineering fields, especially in fatigue life prediction. Machine learning can automatically learn complex nonlinear relationships from a large amount of experimental data, and compared with traditional methods, it has stronger adaptability and prediction ability. These methods can handle high-dimensional complex data and automatically identify key factors affecting the fatigue life of welding joints. In recent years, studies have shown that machine learning algorithms such as the Support Vector Machine (SVM), Random Forest (RF), and the Artificial Neural Network (ANN) have demonstrated significant advantages in predicting the fatigue life of welding joints. For example, Liu et al. [5] used the Random Forest algorithm to model the fatigue life of welding joints, and the results showed that this method performed excellently in predicting the fatigue life of high-strength steels and could effectively improve prediction accuracy. Wu et al. [6] conducted a multi-level analysis of the fatigue life of spot-welding joints based on an Artificial Neural Network model, proving that neural networks can fit complex nonlinear relationships. Especially in the presence of welding defects, their prediction accuracy is better than that of traditional structural stress methods. In addition, domestic scholars have also made some important progress in this field. Liu et al. [7] utilized Random Forest and LightGBM algorithms to predict the fatigue life of welded joints, showing good performance across various geometries and materials.

Moreover, the influence of compressive residual stresses on fatigue life under cyclic loading should also be mentioned. Compressive residual stresses can significantly enhance the fatigue life of machine elements by reducing the effective stress amplitude experienced by the material. For example, in a study on roller bearings, it was found that inducing compressive residual stresses through innovative production processes, such as hard turning combined with deep rolling, can increase the L10 fatigue life of roller bearings by a factor of 2.5 [8]. This highlights the importance of considering residual stresses in fatigue life prediction and provides a valuable reference for improving the fatigue performance of spot-welding joints.

This study aims to propose a new method for predicting the fatigue life of spot-welding joints by combining traditional structural stress methods with machine learning techniques. By analyzing the fatigue test data of Q&P980 steel spot-welding joints and combining multiple machine learning algorithms (such as Random Forest, an Artificial Neural Network, and Gaussian Process Regression), fatigue life prediction models for galvanized and non-galvanized steels were established. The experimental results showed that machine learning methods outperformed traditional methods in terms of prediction accuracy and generalization ability, especially in predicting the fatigue life of high-strength and galvanized steel welding joints, where machine learning models could significantly improve prediction accuracy. This study provides an effective tool for the fatigue life assessment of spot-welding joints and highlights the potential application of machine learning in this field.

## 2. Fatigue Testing of Q&P980 Steel Spot-Welded Joint

### 2.1. Fatigue Test Scheme

#### 2.1.1. Preparation of Fatigue Specimens

**Figure 1:**
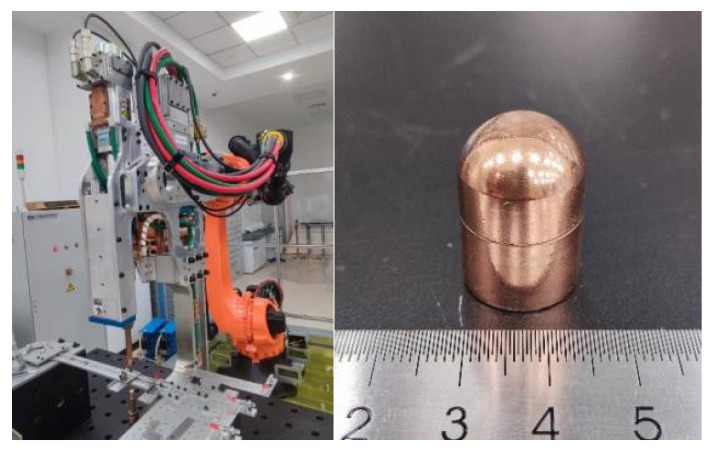
Resistance spot-welding equipment and electrode shape.

The resistance spot-welding equipment used in the experiment is driven by a KUKA robot (KUKA AG, Augsburg, Germany) and features an OBARA medium-frequency inverter DC welding gun (OBARA Corporation, Osaka, Japan). This equipment has a maximum stroke of 200 mm, a maximum welding current of 40,000 A, and a maximum electrode pressure of 6000 N. The accuracy of both the output current and pressure is ±0.1. The equipment is also equipped with a recirculating water-cooling system to ensure a stable welding process. Figure 1 shows the appearance of the resistance spot-welding experimental setup and the electrode tip. The electrode tip used is a standard F1-16-20-8-6.5 type Cu-Cr electrode tip, with an electrode cross-sectional dimension of 6 mm.

**Figure 2:**
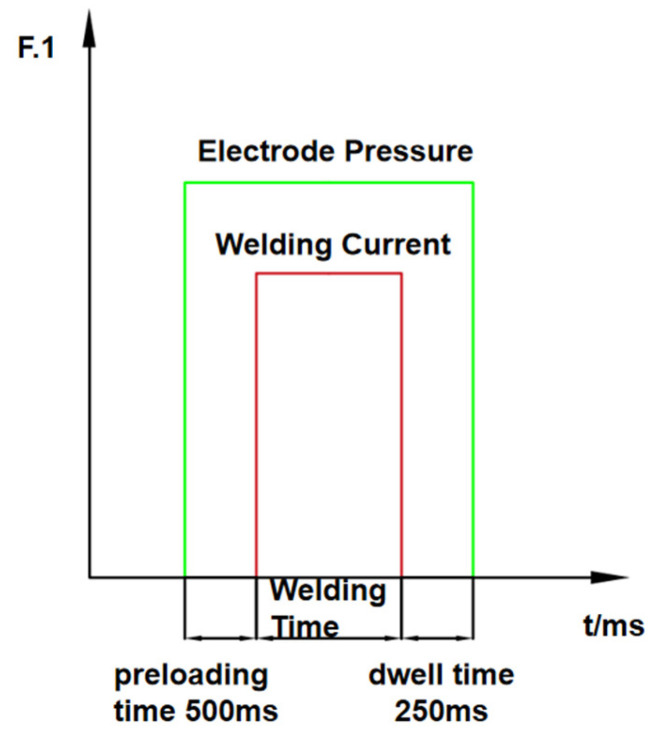
Welding conditions for Q&P980 steel resistance spot welding.

Referring to relevant industry handbooks and standards, the process parameters were determined. The selected process parameters are as follows: welding current of 10 kA, welding time of 360 ms, and electrode force of 3 kN. To minimize random errors, each type of sheet was subjected to three repeated welding tests under these parameters. The single-pulse welding method was employed in this experiment, as shown in Figure 2.

The electrode indentation into the welded material was controlled by the welding pressure and time. The typical indentation depth was approximately 0.5 mm. The thickness of the welded sheets used in the fatigue tests was 1.2 mm. The thickness of the zinc coating on the galvanized sheets was 13 µm. The weld nugget diameter measured in the fatigue tests was approximately 6.35 mm for galvanized samples and 6.99 mm for non-galvanized samples. The nominal weld nugget diameter was determined based on the average measured diameter from multiple samples, which is a common practice in the industry to ensure the consistency and reliability of the welding process. Specifically, the nominal weld nugget diameter was calculated using the formula:

(1) $$D_{nugget}=3.5\times \sqrt{t}$$

where t is the thickness of the welded sheet.

#### 2.1.2. Design of Fatigue Experiments

This study further extends the fatigue testing research on Q&P980 steel spot-welding joints based on existing studies. The test scheme for the TS specimen referenced the relevant literature [2]. On this basis, this study conducted a thorough analysis of the CP specimen, focusing on the impact of the galvanized layer versus the non-galvanized layer on the fatigue life of spot-welding joints, forming a contrast with the research on TS specimens.

**Figure 3:**
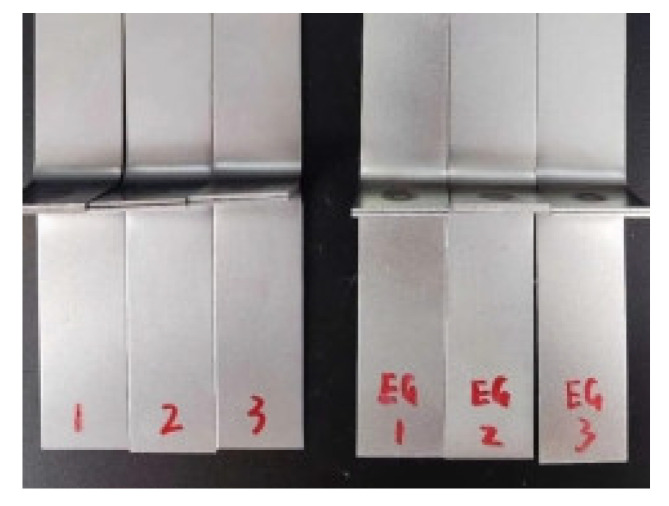
Fatigue test specimen of CP.

Fatigue testing was conducted at room temperature using a constant amplitude load control method. In this method, the maximum and minimum loads applied in each cycle are kept constant until failure occurs. The number of cycles at failure is defined as the fatigue life. To save economic and time costs, load amplitude tests were performed for the CP specimens for comparison. To avoid accidental results in the tests, each group was repeated three times, as shown in Figure 3 (the left side shows the non-galvanized specimens and the right side shows the galvanized specimens).

For the CP specimens, the load ratio R = 0.1, defined as the ratio of minimum to maximum loads during cyclic loading, was used for both the galvanized and non-galvanized samples. This R ratio is consistent with that used in previous studies [2]. Due to the lower stiffness of the CP specimens, which can cause significant vibration, the testing frequency for the CP specimens was set at 10 Hz. If no fatigue failure occurs after reaching a total of 106 cycles in the fatigue test, the load is defined as the fatigue limit.

### 2.2. Fatigue Test Results

The fatigue test results indicate that the galvanized layer has a significant impact on the fatigue life of spot-welding joints. The fatigue test results of the TS specimen are consistent with the study in Reference [2], showing that the fatigue life of the galvanized specimen is significantly lower than that of the non-galvanized specimen. This is mainly due to the generation of LME (Liquid Metal Embrittlement) cracks caused by the galvanized layer, which significantly affects the fatigue performance of the joint. In contrast, the results of the newly added CP specimen in this study show that under the CP loading mode, the difference in fatigue life between galvanized and non-galvanized specimens is relatively small, indicating that LME cracks have a lesser impact on the fatigue life under the CP mode.

Table 1 presents a comparison of the fatigue test results for TS and CP specimens. The data indicate that under the loading conditions of TS specimens, the average fatigue life of spot-welded joints in galvanized specimens is 20,892 cycles, which is significantly lower than the average fatigue life of 41,403 cycles for non-galvanized specimens, representing a nearly 50% reduction in fatigue life. For CP specimens, the average fatigue life of spot-welded joints in galvanized specimens is 29,486 cycles, while that in non-galvanized specimens is 26,916 cycles, showing that the fatigue lives of the two types of joints are quite similar. A preliminary conclusion can be drawn that LME cracks have a certain impact on the fatigue life of spot-welded joints under the TS loading mode but have almost no effect under the CP loading mode, indicating that the influence of LME cracks on fatigue life is relatively small in this mode.

### 2.3. Fatigue Fracture Analysis

#### 2.3.1. Fatigue Fracture of CP Specimens

**Figure 4:**
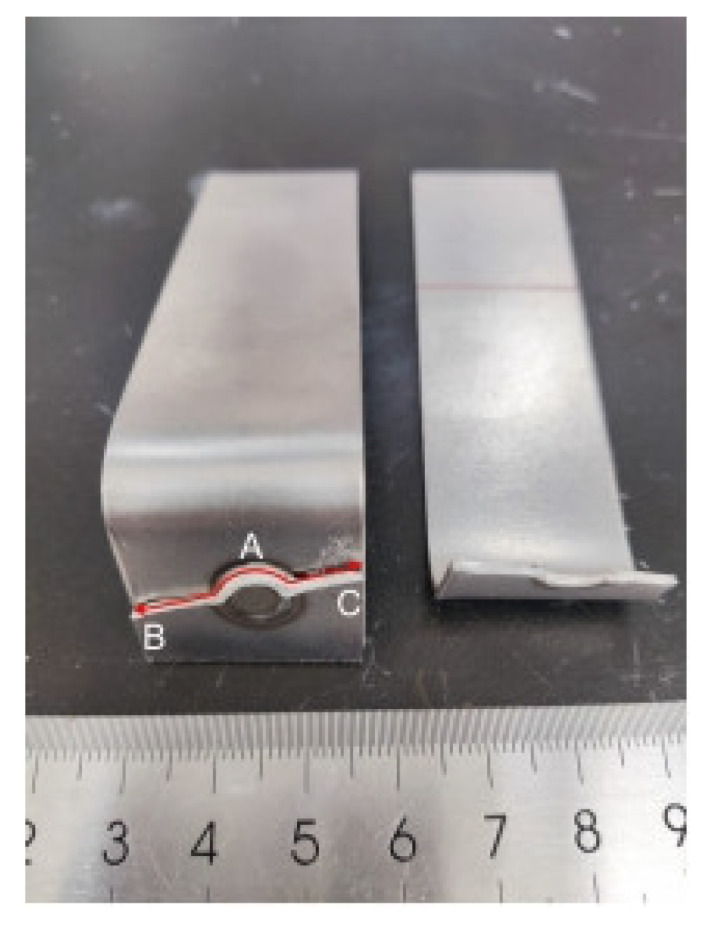
Fracture failure image of the non-galvanized specimen after fatigue testing.

In the fatigue tests described above, the main failure mode occurring in the galvanized specimens in the CP specimens was eyebrow fracture, and there were also some cases of button fracture. The failure mode that occurred in the non-galvanized specimens was eyebrow fracture. The fracture failure image for the fatigue test of the non-galvanized specimen is shown in Figure 4.

**Figure 5:**
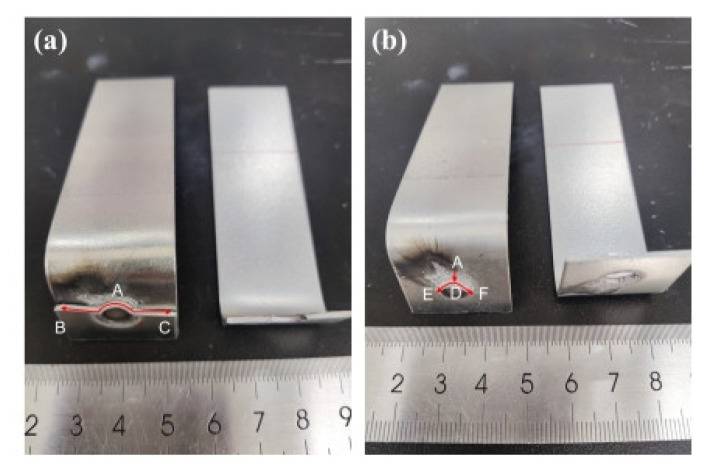
Fatigue fracture failure of galvanized specimen: (**a**) eyebrow fracture; (**b**) partial button fracture.

For the galvanized specimens exhibiting eyebrow fractures, as shown in Figure 5, the fatigue crack was initiated at the high-stress notch on the side subjected to the external force (location A). The fatigue crack propagates through the plate thickness and extends in a direction perpendicular to the tensile stress at locations B and C. No LME cracks were observed during the entire crack propagation process until complete failure occurred.

In contrast, for the galvanized specimens experiencing partial button fractures, the fatigue crack was also initiated at the high-stress notch (location A). Initially, the fatigue crack propagates along the fusion nucleus interface toward location D. After the sheet undergoes deformation, the crack propagation direction changes and begins to extend toward the plate width. Due to the presence of deep LME cracks in the heat-affected zone along the crack propagation path, the crack is forced to propagate along the LME crack circumference at locations E and F until the entire failure process is completed. The fatigue failure process of the non-galvanized specimens is consistent with the eyebrow fracture observed in the galvanized specimens.

**Figure 6:**
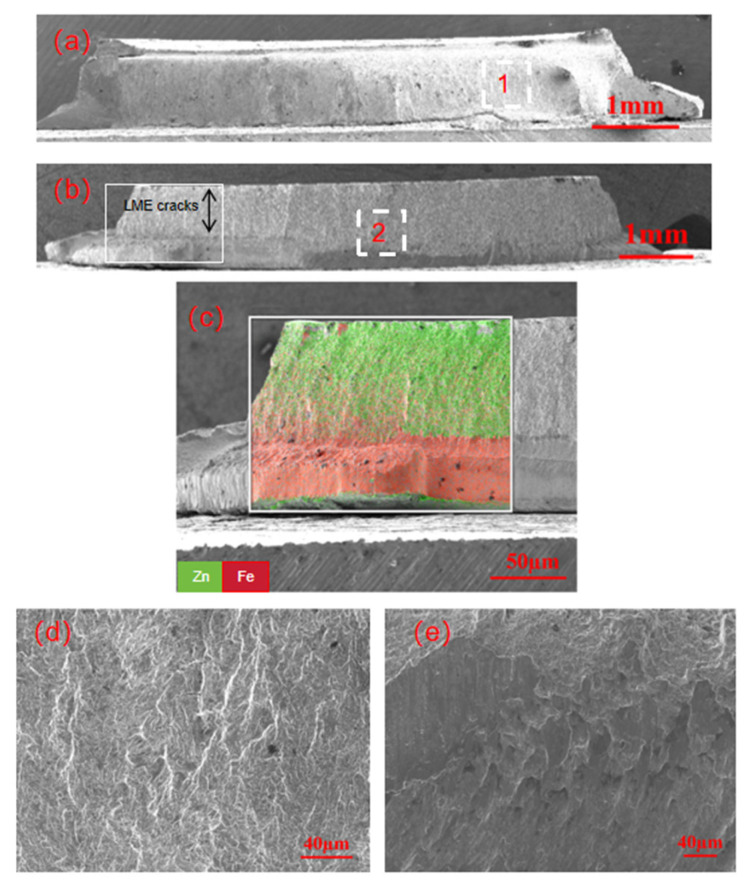
Fatigue test fracture of zinc-plated specimen with partial button fracture: (**a**) overall fracture morphology; (**b**) rear view of the overall fracture morphology; (**c**) elemental scan of the regional energy spectrum; (**d**) local magnification of position 1; (**e**) local magnification of region 2.

Figure 6 shows the fracture appearance of the galvanized specimen undergoing partial button fracture. Figure 6a,b presents the overall microstructure and the rear view, respectively. Figure 6c shows the energy spectrum elemental scan of the region, revealing that the LME crack is deep and essentially penetrates the entire plate thickness. Figure 6d,e shows the micro-enlarged views of front area 1 and rear area 2, respectively. Area 1, located at the center of the fusion nucleus region, exhibits distinct tearing ridges, while area 2 on the back side shows evident shear slip lines. The entire process is characterized as brittle fracture. Due to the presence of a large area of LME cracks, this region mainly serves as the instantaneous fracture zone of the fatigue crack, which has a relatively minor impact on the overall fatigue life and crack propagation.

**Figure 7:**
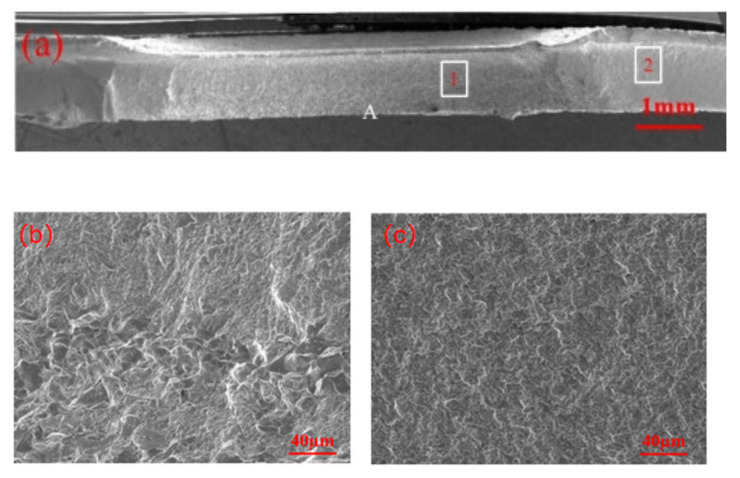
Fatigue test fracture of zinc-plated specimen undergoing eyebrow fracture: (**a**) overall fracture morphology; (**b**) local enlargement of position 1; (**c**) local enlargement of area 2.

Figure 7 shows the fracture appearance of the galvanized specimen undergoing partial eyebrow fracture. Location A in the figure is where the fatigue crack initiates. In area 1, in addition to the distinct tearing ridges, an intergranular fracture is also observed. The tearing ridges in area 2 are smaller in comparison to those in area 1, indicating that the remaining life at this stage is primarily the crack propagation life.

**Figure 8:**
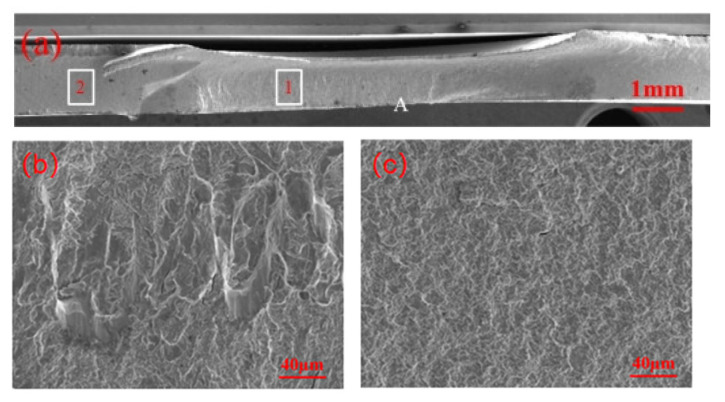
Fatigue fracture of zinc-free specimen: (**a**) overall fracture morphology; (**b**) local enlargement of position 1; (**c**) local enlargement of area 2.

Figure 8 presents the fatigue fracture of the non-galvanized specimen. The crack initiates at the high-stress concentration point (A) and propagates with tearing ridges in region 1 and a tighter crack extension in region 2. The direction of crack propagation aligns with the applied tensile stress.

#### 2.3.2. Summary of Fatigue Fracture Analysis

Through the analysis of the fatigue fracture surfaces of samples with and without galvanizing under different lap joint configurations, significant differences in crack characteristics and failure modes were observed. In the TS [2] specimens, fatigue cracks typically initiate at the high-stress notch tip and propagate perpendicularly to the tensile stress direction. However, in the galvanized specimens, liquid metal embrittlement (LME) cracks alter the crack propagation path, causing the fatigue cracks to pass through the LME crack zones, thereby accelerating the crack propagation and significantly reducing the fatigue life of the spot-welded joint.

In contrast, the fatigue fracture analysis of the CP specimens reveals more complex crack behavior. The fatigue crack propagation path in non-galvanized specimens is relatively regular, while the galvanized specimens exhibit multiple failure modes, including eyebrow fractures and partial button fractures. In the case of partial button fractures, the fatigue crack propagation is influenced by the LME crack zones, but these cracks are mainly located in the instantaneous fracture zone and have a relatively smaller impact on the overall fatigue life of the joint.

Overall, the comparative analysis of the fatigue fracture surfaces of TS and CP specimens indicates that the influence of the galvanized layer on crack propagation paths and failure modes varies with the lap joint configuration. The study of CP specimens further supplements the understanding of the galvanizing effect, especially under the coexistence of multiple failure modes, providing a more comprehensive reference for optimizing welding processes and designing more reliable welded structures.

## 3. Fatigue Data Analysis and Preprocessing

### 3.1. Data Collection

The fatigue life of welded body joints plays a key role in vehicle safety. By predicting the fatigue life of welded joints, the safety of the body structure under different working conditions can be effectively evaluated so as to improve the safety performance of the whole vehicle. This chapter first combines literature and experimental data to pre-process the collected data. Then, a sensitivity analysis is performed to identify the key features affecting the fatigue life of spot-welded joints. Finally, a fatigue life prediction model is established based on the structural stress method and the machine learning method, and the two models are compared and validated.

The fatigue data of spot-welded joints used in this paper are mainly derived from two sources: the related research literature [9,10,11,12,13,14,15,16,17,18,19] and the experimental data obtained from the fatigue tests described above, both of which are based on steel materials. The fatigue life of spot-welded joints is affected by a variety of factors, its control mechanism is more complex, and there is a certain degree of uncertainty. Based on the discussion of fatigue life influencing factors in existing studies, this paper takes all the factors that may affect the mechanical properties of spot-welded joints as the initial input features, while the fatigue life of the joints is taken as the output features, as shown in Table 2. The input features are listed in the table, including material properties (tensile strength, yield strength, and elongation), plate dimensions (plate width and plate thickness), joint characterization (fusion core diameter), loading mode (stress ratio), and joint stresses, with some of the characterization data distinguishing between upper and lower plates. The fatigue life dataset totaled 491 sample data.

**Figure 9:**
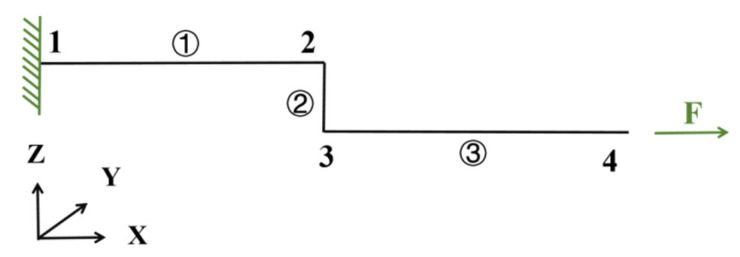
TS specimen beam system.

The variation in loads applied to the joints is an important factor that directly affects the performance of the joints, and according to this study, the forces and moments applied to the spot-welded joints in Table 2 can be derived by numerical calculation methods, with the schematic diagram of the calculation of the structural loads of the joints shown in Figure 9 [20]. This structure consists three beams: beam 1 and beam 3 are deformable and elastic, and beam 2 is rigid, by assuming that the overlap part can be ignored. By substituting the relevant parameters into the calculation formula, the joint loads for each sample’s data can be obtained.

The loads and moments transmitted by the TS specimen joints are calculated as follows:

(2) $$F_{x}=F$$

(3) $$F_{z}=-\frac{12L_{2}}{L_{1}\left(14+\frac{E_{1}I_{1}}{E_{3}I_{3}}+\frac{E_{3}I_{3}}{E_{1}I_{1}}\right)}$$

(4) $$M_{1}=\frac{\left(7+\frac{E_{1}I_{1}}{E_{3}I_{3}}\right)FL_{2}}{14+\frac{E_{1}I_{1}}{E_{3}I_{3}}+\frac{E_{3}I_{3}}{E_{1}I_{1}}}$$

(5) $$M_{2}=\frac{\left(7+\frac{E_{3}I_{3}}{E_{1}I_{1}}\right)FL_{2}}{14+\frac{E_{1}I_{1}}{E_{3}I_{3}}+\frac{E_{3}I_{3}}{E_{1}I_{1}}}$$

where:

*L_i_*—the length (m) of the ith beam.

*E_i_*—modulus of elasticity of the ith beam (Mpa).

*I_i_*—moment of inertia of the ith beam (Kg*m^2^).

*F*—externally applied load (N).

*F_x_* and *F_z_*—the forces (N) applied in the x and z directions.

*M*_1_ and *M*_2_—moments transmitted by the plates (N*m), respectively.

### 3.2. Data Pre-Processing

#### 3.2.1. Data Normalization

For traditional structural mechanics methods, fitting can usually be performed directly by formulas, which involve fewer data features and therefore do not need to process the input data. In machine learning methods, on the other hand, when data with different features are aggregated, the differences in the expression of the features may result in the small data being overwritten by the large data in absolute terms. To ensure that each feature is treated equally, the input data must be normalized. The main purpose of this process is to convert features with different magnitudes to the same magnitude, attenuating the effect of features with large variances on model training, thus improving the accuracy of the model. In addition, the normalization process also helps to speed up the convergence of the neural network model. After normalization, the values of each feature will be controlled in a small range, avoiding the huge differences caused by different magnitudes.

Due to the small dataset used in this paper, the Min-Max normalization method, also known as divergence normalization, was chosen. This method maps the values of each feature to the interval [0, 1] through a linear process. Its transformation formula is:

(6) $$X_{\prime }=\frac{X-X_{min}}{X_{max}-X_{min}}$$

where:

X and $X_{\prime }$—the original data value and the value after normalization, respectively.

$X_{max}$ and $X_{min}$—the maximum and minimum values in the original dataset, respectively.

Since the output parameter fatigue life is of the order of magnitude between 10^2^ and 10^7^, using logarithmic nonlinear normalization:

(7) $$Y=In(N_{f})$$

where:

*Y*—standardized fatigue life (cyc).

*N_f_*—the true fatigue life (cyc) in the original dataset.

The fatigue life output from the model is processed using inverse normalization to obtain the original fatigue life predicted by the model.

#### 3.2.2. Dataset Division

In this paper, the dataset is divided into two mutually exclusive subsets, one set as the training set and the other set as the test set. To achieve this division, the equal interval sampling method is used. Equal interval sampling is a simple and intuitive way to ensure a relatively balanced number of samples within each division interval by selecting the samples uniformly, which helps to reduce the sample selection bias and thus improves the generalization ability of the model. In addition, equal-interval sampling is deterministic, and as long as the interval size and the starting point of the division remain consistent, it ensures the reproducibility of the results and facilitates subsequent comparison and validation. Specifically, in this paper, one sample is taken every five samples as the test set data, and the rest of the samples are used in the training set, of which 393 are in the training set and 98 are in the test set, with a sample data ratio of 4:1.

## 4. Fatigue Life Prediction Methods for Spot-Welded Joints

### 4.1. Theoretical Empirical Formulas

In the introduction, we briefly introduced the traditional structural stress methods and their limitations in predicting the fatigue life of spot-welding joints. This section will provide a detailed description of these methods, including the specific calculation process and relevant formulas.

#### 4.1.1. Rupp Structural Stress Method

**Figure 10:**
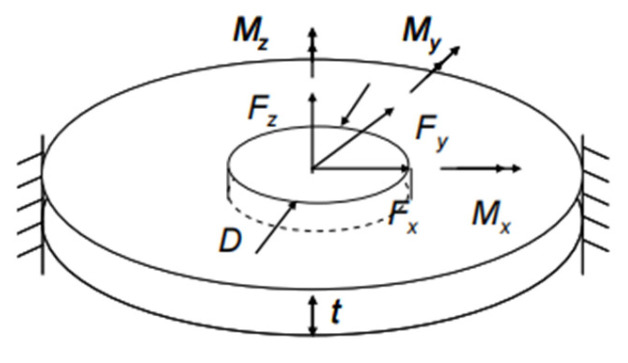
Schematic of the Rupp disk stress model.

The fatigue life prediction of the weld point can be achieved through the structural stress method. In 1995, Rupp et al. [3] proposed a classical model that regards the weld point as a disk model, as shown in Figure 10. By calculating the forces and moments in the three principal stress directions in the local coordinate system of the weld point, the structural stress can be determined. In this model, the area on the circumference of the weld point with the largest stress variation is a potentially hazardous area. Using the range of structural stress variation in this area as a fatigue control parameter, the fatigue life of the spot-welded joint can be further predicted.

Through a large number of experimental observations, it was found that usually the failure location of the welded joint is located in the peripheral region of the weld core, so the structural stress is introduced into the amplitude angle on the circumference of the welded joint θ to calculate the equivalent peripheral structural stress, which is calculated by the following formula [21]:

(8) $$S_{eq}=-S_{max}\left(F_{x}\right)cos\theta -S_{max}\left(F_{y}\right)sin\theta +S_{max}\left(F_{z}\right)\;+S_{max}\left(M_{x}\right)sin\theta -S_{max}\left(M_{y}\right)cos\theta$$

Among them:

(9) $$S_{max}\left(F_{x}\right)=\frac{F_{x}}{\pi Dt}$$

(10) $$S_{max}\left(F_{y}\right)=\frac{F_{y}}{\pi Dt}$$

(11) $$S_{max}\left(F_{z}\right)=k\left(\frac{1.744F_{z}}{t^{2}}\right)\mathrm{for}\;F_{z}>0$$

(12) $$S_{max}\left(F_{z}\right)=0\mathrm{for}\;F_{z}\leq 0$$

(13) $$S_{max}\left(M_{x}\right)=k\left(\frac{1.872M_{x}}{Dt^{2}}\right)$$

(14) $$S_{max}\left(M_{y}\right)=k\left(\frac{1.872M_{y}}{Dt^{2}}\right)$$

where:

*F_x_*, *F_y_* and *F_z_* with *M_x_* and *My*—the load (N) and moment (N*m) applied in the direction of the coordinate axis of the welded joint, respectively.

*S_max_*—the maximum value of the structural stress produced by the corresponding load (Mpa).

*D*—diameter of the weld joint nucleus (mm).

*t*—plate thickness (mm).

*k*—a material-dependent constant, usually taken as $0.6\sqrt{t}$, as compensation for bending stress gradient effects.

#### 4.1.2. Rupp-Modified Structural Stress Method

To improve the accuracy of fatigue life prediction, Yang et al. introduced a mean stress correction factor M to the calculation of the equivalent stress amplitude S_0_, as shown in the following formula:

(15) $$S_{0}=\frac{S+MS_{m}}{M+1}$$

where:

*S*—structural stress.

*M*—average stress sensitivity factor, usually taken as M = 0.1.

*S_m_*—mean stress.

#### 4.1.3. Empirical Formulas for Fitting Curves

By calculating the equivalent stress amplitude range ∆S, which is determined by considering the maximum and minimum stress values experienced by the material under cyclic loading, and then finding the range between these values and fatigue life N_f_ fitting to obtain a ∆S-N_f_ curve, known as the approximate main S-N curve, the fatigue test data obtained will be in a small range above and below the curve and the assessment of the fatigue life of the spot-welded head can be accomplished by using this curve. The fitting equation is:

(16) $$∆S=SRI{\left(N_{f}\right)}^{b}$$

where:

∆S—structural stress range values (Mpa).

*SRI*—curve stress range intercept (MPa).

*N_f_*—fatigue life (cyc).

*b*—slope of the curve.

### 4.2. Machine Learning Algorithms

Machine learning is an intersectional field involving several disciplines, covering knowledge of computer science, probability theory, statistics, and approximation theory. Its essence is that through a large amount of data and certain algorithmic rules, computers are able to simulate the human learning process, continuously improve their performance through continuous data learning, and ultimately make intelligent decisions [22]. In the process of machine learning, data constitute the core driving force, and algorithms are trained on these data to reveal the underlying laws. Choosing the right algorithm, as well as the structure and parameters of the model, will directly affect the accuracy of the model [23]. Common machine learning algorithms include Support Vector Machines (SVMs), Artificial Neural Networks (ANNs), Gaussian Process Regression (GPR), Random Forests (RFs), K-Nearest Neighbors (KNNs), and so on. Several of the machine learning algorithms used in this section are briefly described below.

#### 4.2.1. Artificial Neural Network Algorithm

The Artificial Neural Network (ANN) is a mathematical or computational model that mimics the structure and function of biological neural networks and is widely used in machine learning and cognitive science. It was first proposed by McCulloch and Pitts in 1943 and has been widely used in various prediction tasks since then. Compared with the traditional single-layer perceptron that can only handle simple linearly differentiable problems, modern Artificial Neural Networks are usually composed of multiple layers of perceptrons and are capable of solving complex problems that cannot be handled by single-layer perceptrons, such as the heteroscedastic problem.

A typical artificial neural network consists of three main layers: an input layer, an output layer, and a hidden layer. Neurons in the input layer receive data inputs and the output layer provides the final prediction, while neurons in the hidden layer are located between the input and output layers and are responsible for receiving outputs from the previous layer and generating inputs for the next layer. The neural network transmits signals through the connections of these neurons, thus enabling the learning and mapping of data.

ANNs usually consist of a large number of nonlinear neurons that are tightly connected to each other with distributed storage properties. The contribution of each neuron to each connection is small, so the neural network has a certain degree of fault tolerance, and a small number of failures usually do not significantly affect the overall performance. The process of implementing an Artificial Neural Network consists of three phases: network parameter selection, training, and testing. Common types of neural networks include the Radial Basis Function Network (RBF), the Recurrent Neural Network (RNN), and Back Propagation (BP) neural networks.

**Figure 11:**
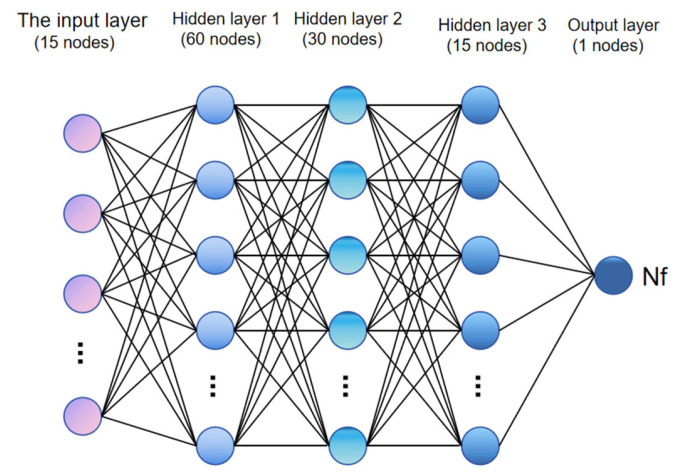
Neural network structure diagram.

In this paper, a BP neural network model is used, and the network structure is set as follows: the input layer contains 15 nodes, the 3 hidden layers contain 60, 30, and 15 nodes, respectively, and the output layer contains 1 node. A dropout layer is added to each hidden layer of the network, and 40%, 30%, and 20% of the neurons are discarded to prevent overfitting. The optimizer is “Adam”, the activation function is “ReLU”, the loss function is the mean square error (MSE), and the number of training iterations is set to 500. The structure of the neural network is shown in Figure 11.

#### 4.2.2. Gaussian Process Regression Algorithm

Gaussian Process Regression (GPR) is a nonparametric machine learning method that can effectively deal with nonlinear and multidimensional parametric problems with small sample data [24]. The core of the GPR algorithm is based on multivariate Gaussian processes and Bayesian inference to model nonlinear problems. We assume that a dataset with a finite sample space exists, where $X={\left[x_{1},x_{2},\ldots x_{n}\right]}^{T}$ is an n-dimensional matrix and $x_{i}\epsilon R^{k}$ is a k-dimensional input vector. $y_{i}\epsilon R$ is the corresponding output vector and YD is the corresponding output vector. The regression process is the nonlinear mapping relationship between input X and output YD, which will be learned based on the sample set, and this regression model is formulated as:

(17) $$y=f\left(x_{*}\right)+\varepsilon$$

where:

y—the observed value after considering noise.

f—no noise predictions are considered.

$x_{*}$—input vector.

ε—noise.

When $\varepsilon \sim N(0,\sigma_{n}^{2})$, a noise of 0 indicates that the GPR is fully interpolated to the original sample, so $\sigma_{n}^{2}$ prevents the regression from overfitting.

A multivariate Gaussian process can be defined such that any input variable x $x\epsilon R^{d}$ satisfies the Bayesian prior distribution of the Gaussian process

(18) $$f\left(x\right)\sim GP(m\left(x\right),k(x,x’))$$

where:

$m\left(x\right)$—mean function.

In practice, it is usually set to 0 to simplify the solution of the posterior probability distribution. $k(x,x_{\prime })$ is the kernel function.

The most commonly used kernel functions for GPR are the linear kernel function, radial basis kernel function, Matern kernel function, quadratic rational kernel function, and so on.

In this paper, the Matern kernel function is used, the control smoothing degree parameter is set to 1.7, the optimization hyperparameter is set to 9, and the noise variance is 0.6.

#### 4.2.3. Random Forest Algorithm

**Figure 12:**
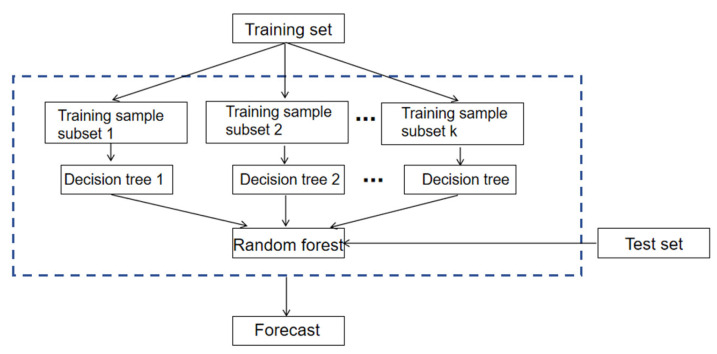
Random Forest algorithm schematic.

The Random Forest (RF) algorithm is an integrated learning method based on decision trees with the advantages of simplicity, low computational overhead, no overfitting, and ease of implementation. RF models decision trees by randomly selecting multiple samples from the original data using the Bootstrap resampling technique and combining these decision trees together to form a powerful learning machine. The final regression or classification result is derived by averaging or voting on all the generated decision tree results. This integrated learning approach can effectively combine the advantages of multiple decision trees, thus improving the robustness and generalization performance of the model. The basic framework of the algorithm is shown in Figure 12.

Since RF is a collection of multiple decision trees, it is able to classify and fit data nonlinearly and does not require feature selection, making it highly adaptable to the dataset. It can handle not only discrete data but also continuous data and has a faster training speed, which makes it suitable for large-scale datasets. In addition, since each decision tree can be generated independently and in parallel, RF is easy to parallelize and improve computational efficiency.

In this paper, the Random Forest is configured as follows: the number of decision trees is 300, the minimum number of samples for split nodes is set to 3, the minimum number of samples for leaf nodes is set to 1, and the evaluation metric for cut quality is the mean square error (MSE).

### 4.3. Model Assessment Metrics and Feature Sensitivity Analysis

#### 4.3.1. Model Evaluation Indicators

Both the structural stress method and the machine learning method selected in this chapter require more uniform evaluation metrics to assess the model’s predictive effectiveness.

(1) Mean Absolute Percentage Error (MAPE): MAPE is an important indicator of the accuracy of the predicted values. MAPE is in the form of a percentage, indicating the average percentage of the relative error between the predicted value and the actual value. The value of MAPE ranges from [0, +∞), and the smaller it is, the more accurate the prediction model is. Its formula is as follows:


(19) $$MAPE=\frac{1}{n}\sum \left(\left|\frac{y_{i}-{\hat{y}}_{i}}{y_{i}}\right|\right)*100\%$$

(2) Root Mean Square Error (RMSE): RMSE is the value of the square root of the mean of the squares of the differences between the actual and predicted values and is used as a measure of the difference between the model’s predicted values and the actual observed values, with the formula:


(20) $$RMSE=\sqrt{\frac{1}{n}\sum_{i=1}^{n}\;(y_{i}-{\hat{y}}_{i}{)}^{2}}$$

(3) Coefficient of determination: R^2^ is a statistic that measures the goodness of fit of a regression model, and its value is between 0 and 1. The closer R^2^ is to 1, the better the model fits the data, and the formula is:


(21) $$R^{2}=1-\frac{\sum_{i=1}^{n}\;{\left(y_{i}-\hat{y_{i}}\right)}^{2}}{\sum_{i=1}^{n}\;{\left(y_{i}-\bar{y_{i}}\right)}^{2}}$$

In the above equation:

$y_{i}$—true value.

${\hat{y}}_{i}$—predicted value.

For the three above-mentioned evaluation indexes, when the MAPE value is smaller, the RMSE value is smaller, and the R^2^ value is closer to 1, the model is well-fitted.

#### 4.3.2. Characterization Sensitivity Analysis

Sensitivity analysis is a statistical analysis method used to study the weight or direction relationship between two or more random variables. Numerous features mentioned in the previous section may have an effect on the fatigue life of the spot-welded head, and in order to more intuitively analyze the effect of each feature on the fatigue life, this paper adopts the Sobol algorithm for sensitivity analysis.

The Sobol algorithm is a global sensitivity analysis method based on variance decomposition. It evaluates the importance of each input parameter to the output result by calculating the first-order sensitivity and total sensitivity indices. The objective function of the model is decomposed into a sum of incremental terms, and the importance of each input parameter is quantified by its contribution to the output variance.

The Sobol algorithm decomposes the objective function f(x) into a sum of incremental terms as follows:

(22) $$f\left(x_{1},x_{2},...,x_{n}\right)=f_{0}+\sum_{i=1}^{n}f_{1}\left(x_{i}\right)+\sum_{i=1}^{n}\sum_{j=i+1}^{n}f_{ij}\left(x_{i},x_{j}\right)+\cdots +f_{i....n}\left(x_{i},...,n\right)$$

where:

$f_{0}$—constant in the objective function.

When each integral variable in Equation (22) is 0 (indicating no contribution when all inputs are zero), the expression is:

(23) $$\int_{0}^{1}f_{i1,\ldots ,is}(x_{i1},x_{i2},\ldots ,x_{is})dx_{ik}=0$$

Among them: $1\leq i_{1}<\cdots <i_{s}\leq n$, 1≤k≤n.

Total variance:

(24) $$V\left(Y\right)=\int f^{2}(x)dx-f_{0}^{2}$$

Bias variance:

(25) $$V_{i_{1},\ldots ,i_{s}}=\int f_{i_{1},\ldots ,i_{s}}^{2}dx_{i_{1}}dx_{i2}\ldots dx_{is}$$

First-order sensitivity coefficients:

(26) $$S_{i}=\frac{V_{i}}{V(Y)}$$

Parameter total sensitivity index:

(27) $$S_{Ti}=1-\frac{V_{\sim i}}{V(Y)}$$

where:

V(Y)—the sum of the results of the parameters on the output of the model’s objective function f(x).

$V_{i_{1},\ldots ,i_{s}}$—the effect of the interaction of the parameter combinations on the model output.

$V_{i}$—the effect of the ith parameter on the output results of the model objective function f(x).

$V_{\sim i}$—the sum of variances due to all parameters other than the ith parameter.

In our study, the Sobol algorithm was applied to the entire dataset used for training our models. The importance scores were automatically generated by the algorithm based on the data analysis. These scores provide a quantitative measure of the influence of each input feature on the fatigue life of spot-welded joints.

**Figure 13:**
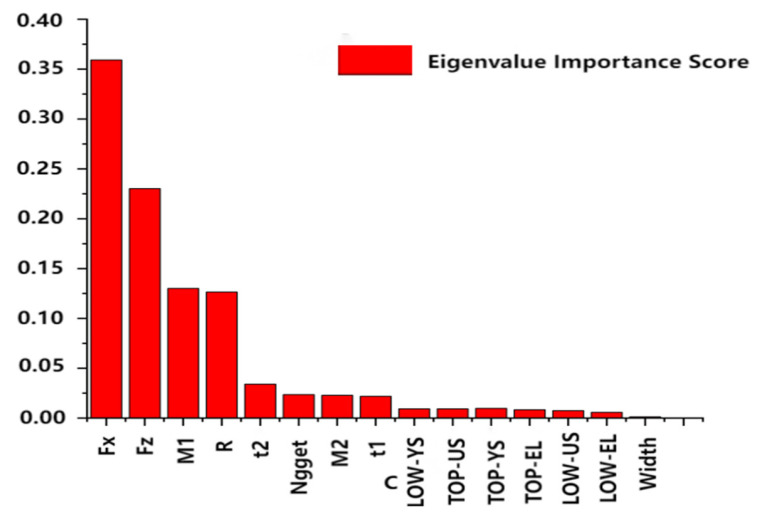
Importance relationship between input features and fatigue life.

Figure 13 illustrates the important relationship between the 15 input features and the output feature (fatigue life). This relationship was obtained through a Sobol sensitivity analysis performed on the entire dataset used for training our models, which includes both the training and validation data. By analyzing the results in the figure, it can be seen that the influence of each input feature on fatigue life varies widely. All 15 input features have a significant impact on the fatigue life of the spot-welded joints and will be used in the subsequent machine learning analysis.

Specifically, according to the Sobol sensitivity analysis, the features with an importance score of more than 0.1 are mainly the external loads on the joint (F_x_, F_m_, F_z_, M_1_ and M_2_) and the stress ratio (R), which, according to the analysis, cause the greatest impact on the joint performance and are the most important influencing factors; the features with an importance score of more than 0.02 include the external loads on the joint (F_x_, F_m_, F_z_, and M_1_), the melt core diameter (Nugget), the plate thickness (t_1_ and t_2_), and the stress ratio (R). Among them, the external loads on the joint (F_x_, F_m_, F_z_, M_1_ and M_2_) are considered to be the key factors directly affecting the performance of the welded joints, and hence their importance scores are high. Molten core diameter (Nugget) and plate thickness (t_1_ and t_2_), on the other hand, are important factors affecting the strength of the joint and have equally high importance scores. The stress ratio (R), on the other hand, influences the amplitude of fatigue loading, thus increasing the risk of fatigue crack initiation and extension, which in turn significantly affects the fatigue life of the joint. However, the material properties of the plate (tensile strength US, yield strength YS, and elongation EL) have a more indirect effect on the fatigue life of spot-welded joints. Since the fatigue life of spot-welded joints is mainly related to the extension life of fatigue cracks, the strength and elongation of the plate have a more significant effect on crack initiation and a lesser effect on the overall fatigue life process. In addition, the small variety and dispersion of plate widths (Width) in the data resulted in a lower significance score.

### 4.4. Analysis of Fatigue Life Prediction Results

#### 4.4.1. Theoretical Empirical Formula Fitting

**Figure 14:**
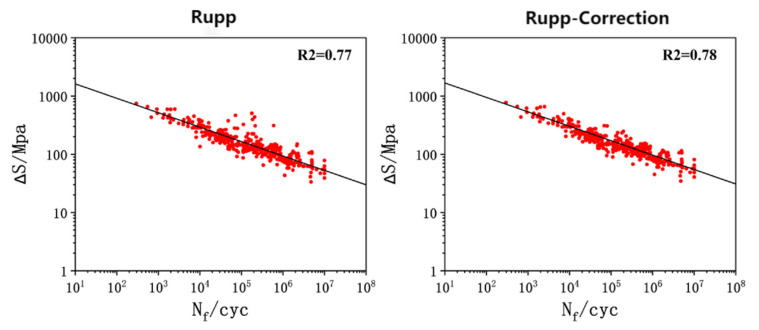
Structural stress method training set formula fitting.

The training set data were fitted using the structural stress method with formulas (Equations (28) and (29)). These formulas were applied to the fatigue test data obtained from systematic experiments on Q&P980 steel spot-welded joints under various loading modes. The fitting was performed using the Simple Fit toolkit in Origin (2022) software. Both the Rupp method and the Rupp method with correction coefficients were employed for comparison. The fitted results are presented in Figure 14. The analysis indicates that the fitting effects of the two methods are similar, with correlation coefficients of 0.77 and 0.78, respectively. The RMSE values for the Rupp method and the Rupp method with correction coefficients are 0.25 and 0.24, respectively. Given these results, the Rupp method with correction coefficients was chosen as the comparison method for subsequent studies. The obtained fitting equations are as follows: Equation (28) represents the Rupp method, while Equation (29) includes the correction coefficients. The calculation results show that the fatigue data points are uniformly distributed around the main S-N curve, indicating a good fitting effect.

(28) $$∆S=2968{\left(N_{f}\right)}^{-0.248}$$

(29) $$∆S=2857{\left(N_{f}\right)}^{-0.247}$$

#### 4.4.2. Analysis of Training Results for the Training Set

Three machine learning algorithms—RF, GPR, and ANN—were employed to train the divided training set data, as previously mentioned. The training results for each algorithm are presented in Table 3. The analysis reveals that all algorithms performed exceptionally well on the training set, achieving correlation coefficients (R^2^) exceeding 0.93 and MSE and MAPE values below 0.1. These results indicate that the machine learning algorithms effectively fit the training set data and are well-suited for application to the test set. Notably, the RF algorithm achieved an R^2^ value of 0.98, surpassing the other two algorithms and demonstrating superior training performance.

#### 4.4.3. Result Discussion

(1) Test Set Prediction Results

**Figure 15:**
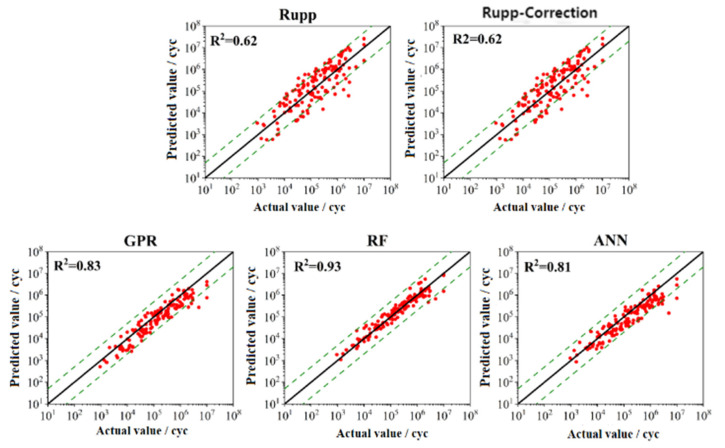
Results based on different training models in the test set.

Predicting the test set data using the trained models allows for a comprehensive assessment of each model’s generalization ability. Table 4 summarizes the performance of each model on the test set. The results show that the traditional structural stress method provided lower predictions, while machine learning algorithms performed better. All machine learning algorithms achieved correlation coefficients exceeding 0.8, with RMSE and MAPE values lower than those of the Rupp and modified Rupp methods. Figure 15 illustrates the fatigue life prediction results for each method on the test set. The structural stress method exhibited more scattered results, with multiple data points failing to fall within the 5-fold error band. In contrast, the machine learning-based algorithms produced more focused results. Although most data points lay within the 5-fold error band, the GPR and ANN algorithms performed less well on the test set than on the training set. The RF algorithm, however, demonstrated strong generalization ability and accurately predicted the fatigue life of the spot-welded joints on both the training and test sets.

(2) Comparative analysis of the prediction results of the optimal model and the structural stress method

Based on the comparative analysis of traditional structural mechanics methods and machine learning algorithms, the Rupp method with correction coefficients and the RF method were selected for predicting the fatigue life of Q&P980 steel spot-welded joints. The test data were derived from fatigue tests on galvanized and non-galvanized samples, with related parameter calculations based on empirical formulas.

**Figure 16:**
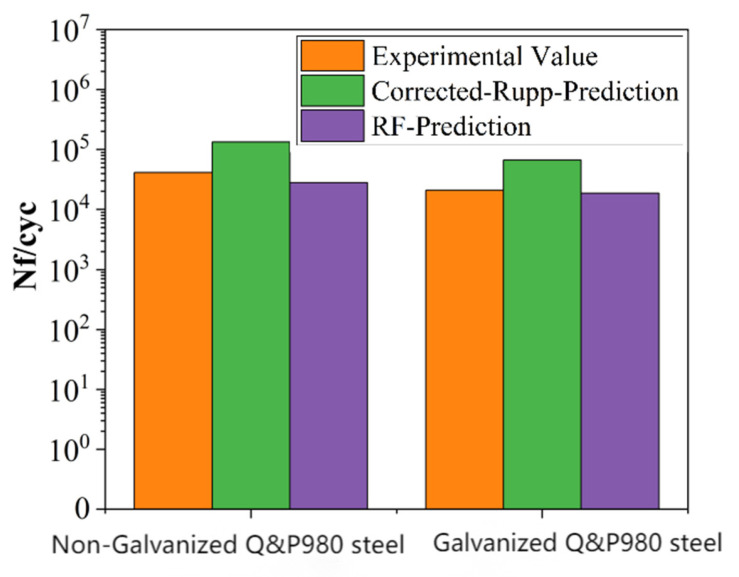
Plot of predicted test results.

The prediction results are shown in Table 5 and visualized in Figure 16. The results indicate that the fatigue life of the non-galvanized specimens is higher than that of the galvanized specimens. Specifically, the RF method provided conservative but accurate predictions: for the galvanized specimen, the RF method predicted a fatigue life of 18,689 cycles, which is 10.2% lower than the actual test result of 20,892 cycles. For the non-galvanized specimen, the RF method predicted a fatigue life of 28,237 cycles, which is 31.8% lower than the actual test result of 41,403 cycles. In contrast, the Rupp-Amendment method was overly optimistic, predicting a fatigue life of 66,688 cycles for the galvanized specimen (219.4% higher than the actual result) and 133,377 cycles for the non-galvanized specimen (221.6% higher than the actual result). This highlights the RF method’s superior predictive accuracy in estimating the fatigue life of spot-welded joints.

The superior performance of the machine learning algorithms, particularly the Random Forest algorithm, highlights the potential of these methods for accurately predicting the fatigue life of spot-welding joints. The ability of machine learning algorithms to handle high-dimensional complex data and automatically identify key factors affecting fatigue life makes them a powerful tool for this application. The results show that machine learning models can significantly improve prediction accuracy, especially in complex engineering problems involving high-strength and galvanized steels.

## 5. Summary

In this paper, the fatigue life of Q&P980 steel spot-welded joints is systematically studied and predicted by combining the traditional structural stress method with machine learning algorithms. Through the processing of fatigue test data, feature sensitivity analysis, and model construction and comparison, the following main conclusions are drawn:

(1) Fatigue test and analysis results:

The effect of the galvanizing layer on the fatigue life of spot-welded joints was investigated through systematic fatigue tests on Q&P980 steel spot-welded joints. The results demonstrated that the galvanizing layer significantly reduces the fatigue life of spot-welded joints. This reduction is primarily attributed to the presence of liquid metal embrittlement (LME) cracks, which alter the crack propagation path and accelerate crack growth. The fatigue life of non-galvanized specimens was found to be higher, indicating that the galvanizing layer has a pronounced negative impact on the fatigue performance of spot-welded joints. Further fatigue fracture analysis revealed that LME cracks influence the crack extension path, providing an explanation for the degradation of the fatigue performance of galvanized joints.

(2) Feature sensitivity analysis:

The effect of different characteristics on the fatigue life of spot-welded joints was investigated by Sobol sensitivity analysis. The results show that the external loads (F_x_, F_z_, M_1_, and M_2_), melt core diameter (Nugget), plate thickness (t_1_ and t_2_), and stress ratio (R) of the joints have a significant effect on the fatigue life, and these characteristics are the key factors affecting the fatigue life of spot-welded joints.

(3) Comparison of machine learning models with traditional methods:

This paper compares the traditional structural stress method (Rupp method) with machine learning algorithms (e.g., Random Forest, Artificial Neural Networks, and Gaussian Process Regression) for fatigue life prediction of spot-welded joints. The experimental results show that the machine learning models, especially the Random Forest (RF) algorithm, exhibit better prediction accuracy than the traditional methods. The RF model outperforms the Rupp method on both the training and test sets, with an R^2^ value of 0.93 and lower RMSE and MAPE values, which indicates that the machine learning algorithms have better prediction and generalization capabilities.

(4) Application prospects of fatigue life prediction:

The machine learning-based prediction model can accurately predict the fatigue life of spot-welded joints, and its prediction results are closer to the actual test data. Compared with the traditional empirical formulas, the machine learning algorithm has a higher prediction accuracy, especially in complex practical engineering. The RF algorithm shows strong practicality and accuracy in fatigue life prediction of galvanized and bare Q&P980 steel, which is suitable for the practical engineering evaluation and design optimization of spot-welded joints.

Future work:

The research presented in this paper demonstrates the advantages of machine learning techniques in fatigue life prediction of spot-welded joints, especially in dealing with complex nonlinear relationships and improving prediction accuracy. In the future, the combination of more engineering data and more advanced machine learning models can further improve the robustness and accuracy of the prediction model and promote the scientific and refined development of the design and life assessment of point-welded joints. Additionally, exploring the application of these methods in other types of welded joints and materials could be a valuable area for future research.

## Figures and Tables

**Table 1:** Comparison of fatigue test results for TS [2] and CP specimens.

Overlap Style	Load Frequency/Hz	Load Amplitude/N	Specimen Number	Galvanized Specimen		Non-Galvanized Specimen	
				Fatigue Life/cyc	Average Lifespan/cyc	Fatigue Life/cyc	Average Lifespan/cyc
TS	30	1800	1	21,053	20,892	50,637	41,403
			2	20,438		40,997	
			3	21,185		32,575	
CP	10	135	1	33,858	29,486	24,895	26,916
			2	25,503		23,149	
			3	29,096		32,705	

**Table 2:** Summary of characterization data.

Feature		Label	Range
Material Properties	tensile strength	US1, US2	306–1355 (Mpa)
	yield strength	YS1, YS2	170–1156 (Mpa)
	elongation	EL1, EL2	5.1–35 (%)
Plate Size	plate thickness	t1, t2	0.8–4 (mm)
	plate width	Width	35–50 (mm)
Joint Characterization	nucleus diameter	Nugget	3.9–13.4 (mm)
Loading method	stress ratio	R	−1~0.3
Joint force (calculated value)	X-direction force	Fx	0–23,837 (N)
	Z-direction force	Fz	7.81–1851 (N)
	Y-direction moment1	M1	493–25,418 (N·mm)
	Y-direction moment2	M2	493–33,541 (N·mm)
fatigue life	fatigue life	Nf	268–107 (cyc)

**Table 3:** Training results for the training set of machine learning algorithms.

Arithmetic	Evaluation Indicators		
	R^2^	RMSE	MAPE
RF	0.98	0.01	0.013
GPR	0.96	0.024	0.017
ANN	0.93	0.026	0.024

**Table 4:** Results for each model in the test set.

Methodologies	Evaluation Indicators		
	R^2^	RMSE	MAPE
Rupp	0.62	0.306	0.096
Rupp-Amendment	0.62	0.305	0.095
RF	0.93	0.056	0.033
GPR	0.83	0.139	0.061
ANN	0.81	0.152	0.06

**Table 5:** Comparison of predicted values for galvanized and non-galvanized specimens.

Specimen Type	Test Result (cyc)	Rupp-Amendment Prediction (cyc)	RF Prediction (cyc)
Galvanized	20,892	66,688	18,689
Non-Galvanized	41,403	133,377	28,237

## Data Availability

The original contributions presented in this study are included in the article. Further inquiries can be directed to the corresponding author.

## References

[1] Kong F.P., Zou S.J. (2024). Fatigue life prediction and maintenance strategies in ship structural design. Ship Mater. Mark..

[2] Zou J., Xiang H., Zhan Z., Zhuo W., Huang L., Ji Y., Liu Q. (2024). Research on the influence of liquid metal embrittlement cracks on the strength and fatigue life of spot-welded joints of galvanized Q&P980 steel. Materials.

[3] Rupp A., Störzel K., Grubisic V. (1995). Computer Aided Dimensioning of Spot-Welded Automotive Structures.

[4] Du W.C. (2021). Microstructure, Mechanical Properties, and Liquid Metal Embrittlement of PHS1500 Galvanized Steel Spot Welded Joints. Master’s Dissertation.

[5] Liu Z., Zhao Y., Wang X. (2020). Fatigue life prediction of welded joints using random forest algorithm. J. Mater. Sci. Technol..

[6] Wu G., Li D., Su X. (2019). Fatigue life prediction of spot welded joints based on artificial neural network. Int. J. Fatigue.

[7] Xu M., Qi H.Y., Li S.L., Shi D.Q., Yang X.G. (2025). Machine-Learning-Based Fatigue Life Prediction Method for Welded Joints. Aeroengine.

[8] Pape F., Maiss O., Denkena B., Poll G. (2019). Enhancement of roller bearing fatigue life by innovative production processes. Ind. Lubr. Tribol..

[9] Zhan L., Qin X.G. (2017). Fatigue life prediction of stainless steel car body side wall resistance spot welded joints. J. Jiamusi Univ. (Nat. Sci. Ed.).

[10] Han X.H., Liu S.L., Zhao Y.Q. (2013). Optimization of fatigue performance of high-stress spot welded joints in car bodies based on nugget diameter. China Railw. Sci..

[11] Long H.Q. (2017). Characteristics of Resistance Spot Welding and Fatigue Crack Propagation of DP590/DC01 Dissimilar Thickness Steel Plates. Ph.D. Dissertation.

[12] Qian T. (2015). Research on Fatigue Life Simulation Prediction Method of High-Strength Steel Weld Points Based on Mean Stress Intensity Factor. Master’s Dissertation.

[13] Wang R. (2022). Deformation of Dissimilar Resistance Spot Welded Joints of Low Carbon Steel and Austenitic Stainless Steel and Its Influence on Mechanical Properties. Master’s Dissertation.

[14] Zhang Z.X. (2016). Fatigue Performance of Weld Points of DP980-GMW2 and Q&P980-GMW2 Steel Plates. Master’s Thesis.

[15] Bonnen J.J., Agrawal H., Amaya M.A., Iyengar R.M., Kang H.T., Khosrovaneh A.K., Link T.M., Shih H.C., Walp M., Yan B. (2006). Fatigue of advanced high strength steel spot-welds. Int. J. Fatigue.

[16] Wu G., Li D., Su X., Peng Y., Shi Y., Huang L., Huang S., Tang W. (2017). Experiment and modeling on fatigue of the DP780GI spot welded joint. Mater. Sci. Eng. A.

[17] Long X., Khanna S.K. (2007). Fatigue properties and failure characterization of spot welded high strength steel sheet. Int. J. Fatigue.

[18] Hu J.H., Song K., Xiong L.M. (2020). Fatigue life study of weld point defects considering the modified mean equivalent stress intensity factor. China Mech. Eng..

[19] Tang J.Z., Zou D.Q., Jiang H.M., Chen X.P., Li S.H. (2014). Experimental study on resistance spot welding process and fatigue performance of Q&P steel lap joints. Hot Work. Technol..

[20] Huang L., Shi Y., Guo H., Su X. (2016). Fatigue behavior and life prediction of self-piercing riveted joint. Int. J. Fatigue.

[21] Yang Y.N., Wang R.J., Yan K. (2016). Prediction of fatigue life of spot welded specimens using structural stress method. Weld. Mach..

[22] Mi X.X., Tang A.T., Zhu Y.C., Kang L., Pan F.S. (2021). Advances in the application of machine learning techniques in materials science. Mater. Rep..

[23] Wang H.W., Ye B., Feng J., Zhong X.Y. (2023). Review on the application of machine learning in steel materials research. China Mater. Prog..

[24] Cheng M., Jiao L., Yan P., Feng L., Qiu T., Wang X., Zhang B. (2021). Prediction of surface residual stress in end milling with Gaussian process regression. J. Manuf. Sci. Eng..

